# Study the seasonal steroid hormones of common carp in Caspian Sea, Iran

**DOI:** 10.1186/2193-1801-2-193

**Published:** 2013-04-30

**Authors:** Vahid Taghizadeh, Mohammad Reza Imanpoor, Nooshin Mehdinejad

**Affiliations:** Department of Fisheries, Gorgan University of Agricultural Sciences and Natural Resources, Gorgan, Iran

**Keywords:** Common carp, *Cyprinus Carpio*, Steroid hormones, Testosterone, 17β-estradiol, Progesterone, Seasons

## Abstract

In this investigation, serum steroid hormones such as testosterone (T), 17β-estradiol (E2) and progesterone (P) in 12 female of the migratory population of Common carp (*Cyprinus carpio*) in southeast of Caspian Sea during a year from May 2011 to May 2012 were studied. The results of present study revealed that changes in levels of steroid hormones, (E2) and (T) were closely correlated to ovarian development. There was significant difference in level of 17 β- estradiol between autumn and winter seasons that the highest of 17-β estradiol level was observed in autumn season. In the case of progesterone hormone, higher levels was recorded in summer season and there was significant difference between summer and spring seasons and lower level of testosterone was observed in spring season.

## Introduction

Sex steroid hormones play important roles in many physiological processes, particularly in the reproduction of vertebrates. In many species of teleost, three sex steroid hormones, 17β-estradiol (E2), 11-ketotestosterone (11-KT) and 17 α20 β- dihydroxy-4-pregnen-3-one (DHP) are abundantly produced in gonadal tissues under the control of pituitary gonadotropins (GTH), and are essential for critical steps of gametogenesis ( Wallace & Browder 1985 ; Agahama & Yamashita 2008 ; Miura et al. 1991 ).

A major estrogen, E2, controls pivotal physiological events in female reproductive cycles in all vertebrates studied to date. The association of changes in gonadal development with plasma levels of gonadal steroids has proven to be a valuable tool for understanding the endocrine control of reproduction in teleosts. Moreover, in teleosts, vitellogenesis and final oocyte maturation are regulated by gonadotropins via steroids secreted by the Granolosa and Theca cells of developing and mature oocytes. The occurrence of steroid production in different cells of the ovary may be related to different phases of oocyte development (Shafiei Sabet et al. 2009 ). Cyclical changes in the reproductive hormones of teleost fishes are widely known to occur in association with reproductive cycles and have been investigated mainly to understand the mechanisms of reproductive behavior, gametogenesis, and gonadal steroidogenesis (Fostier et al. 1983 ; Goetz 1983 ). Seasonal changes in circulating levels of gonadal steroid hormones during the reproductive cycle are described for a variety of freshwater and marine teleost species (Fostier et al. 1983 ; Pankhurst & Carragher 1991 ). With the onset of oocyte maturation/ovulation the estrogen level, which is low in the postvitellogenic ovary, undergoes a further significant reduction in several species including the catfish. *Fossilis* and *Clarias batrachus* (Joy et al. 1998 ) indicating a shift in steroidogenesis.

During vitellogenesis an increase in plasma estrogen levels, mainly estradiol that correlates with the growth of vitellogenic oocytes has been observed in many species. In the tilapia *Sarotherodon aureus* (now *Oreochromis aureus*), the initiation of spawning by increasing water temperature is followed by a rise in testosterone levels (Katz & Eckestein 1974 ). Although it has been ascertained in cyprinids that final oocyte maturation and ovulation are induced by a preovulatory gonadotropin surge, little information on the plasma and gonadal changes in steroid hormone levels during the reproductive cycle in *Cyprinus carpio* is known.

The aim of this work was to investigate the seasonal cycle of the gonadal steroids testosterone (T), 11-ketotestosterone (11-KT), and 17b-estradiol (E2) in the serum of wild-caught populations of *Cyprinus carpio* from the southern Caspian Sea.

## Materials and methods

### Broodstock preparation

The study was conducted between May 2011 and May 2012. 12 specimens of female Common carp were captured from southeast of Caspian Sea during year and transported to Central Laboratory of Gorgan University of Agricultural Science and Natural Resources, Gorgan, Iran. In each season, 3 specimens of female fish were captured. Total weight (937.08 ± 216.5 g) and total length (42.08 ± 3.5 cm) of the fishes were measured.

### Measurement of serum steroid levels

The blood samples were taken from caudal vein with a nonheparinized syringe and centrifuged for 10 min. at 3000 × g, and then serum was stored at -20°C until analyzed.

### Statistical analysis

Data were statistically analyzed by analysis of variance (ANOVA) using the General Linear Models procedure coupled with Duncan’ s multiple range test in SPSS software (Ver. 16.0).

## Results

The mean values and standard deviation of the steroid hormones of *Cyprinus carpio* are summarized in Table 1.

**Table 1:** **Seasonal changes in steroid hormones of*Cyprinus carpio***

Winter	Autumn	Summer	Spring	Seasons
				Hormone
0.46 ± 0.23^ab^	0.62 ± 0.04^ab^	0.71 ± 0.02^a^	0.42 ± 0.01^b^	progesterone
30 ± 14.25^a^	247.73 ± 134.14^a^	110.60 ± 48.27^ab^	110.43 ± 80.13^ab^	17-β estradiol
0.56 ± 0.08^a^	0.71 ± 0.18^a^	0.72 ± 0.02^a^	0.05 ± 0.0^b^	Testosterone

Means with the same superscript letters at the same row are not significantly different (P > 0.05).

Steroid hormones analysis during four seasons showed that there was no significant differences in level of 17β-stradiol among spring, autumn and also between autumn and winter seasons and higher level belong to autumn season. There was significant difference in level of testosterone among spring and autumn seasons. Also level lower of progesterone was observed in summer season.

**Figure 1:**
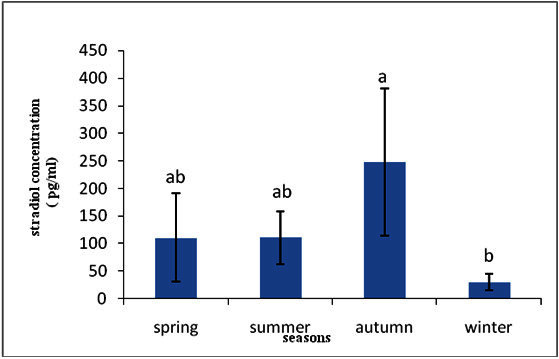
**Stradiol hormone concentration in seasons.**

Level of 17 β, estradiol hormone in spring, summer, autumn and winter seasons were 110.43 ± 80.13, 110.60 ± 48.27, 247.73 ± 134.14 and 30.13 ± 14.25 (Figure 1). There was significant difference in level of 17 β- estradiol between autumn and winter seasons that the highest of 17-β estradiol level was observed in autumn season.

**Figure 2:**
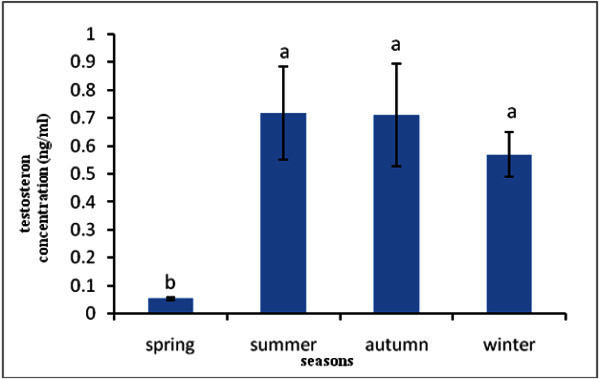
**Testosterone hormone concentration in seasons.**

In the present study level of testosterone hormone in spring, summer, autumn and winter seasons were 0.05 ± 0.005, 0.72 ± 0.02, 0.71 ± 0.18 and 0.56 ± 0.08 that higher level belong to summer season and there was significant difference in level of testosterone among spring season with summer, autumn and winter seasons (Figure 2). Also lower level of testosterone was observed in spring season.

**Figure 3:**
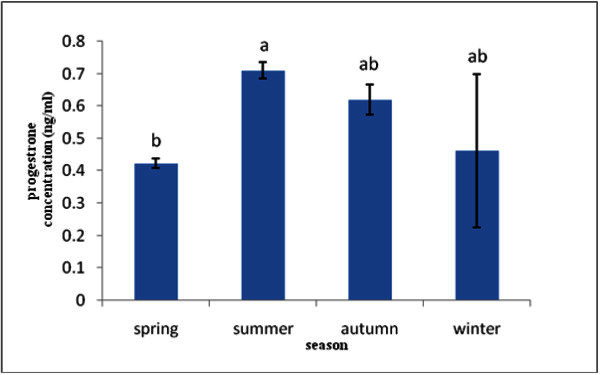
**Progesterone hormone concentration in different seasons.**

In the case of progesterone hormone, higher levels were seen summer season and there was significant difference between summer and spring season. Levels of progesterone hormone in spring, summer, autumn and winter seasons were 0.42 ± 0.01, 0.71 ± 0.02, 0.62 ± 0.04 and 0.46 ± 0.23 (Figure 3).

## Discussion

Three sex steroid hormones, 17β-estradiol (E2), 11-ketotestosterone (11-KT), and 17α, 20β, dihydroxy- 4-pregnen-3-one (DHP) are well established as primary estrogen, androgen, and progestin, respectively, in teleost fish. In vitro and in vivo assays suggest that 11-KT and E2 play primary roles in previtellogenic and growth of oocytes, respectively, whereas DHP is essential for induction of final oocyte maturation (Kazeto et al. 2011 ).

17-β estradiol (E2) is secreted by both the female gonads and inter-renal tissues. In general, estradiol is responsible for stimulating vitellogenesis and is also secreted by female gonads during the pre-spawning period. Evaluation of the results in Table 1 and Figure 1 shown that there is an increase in the level of 17 β-estradiol in autumn season. Estradiol is known to be secreted by the cells of the ovarian follicles that promote the development and maintenance of the female sexual characteristics. In humans this hormone (together with other hormones) is responsible for controlling the female sexual cycle. Estradiol has been reported to stimulate vitellogenesis in teleosts (Campbell & Idler 1976 ; De Vlaming et al. 1980 ; Smith & Haley 1988 ). These authors have reported an increase in plasma estradiol levels once spawning commences, and that it remains high throughout the period of oocyte growth. These observations suggest that during this phase of undetectable estradiol levels, no vitellogenesis is required during the mouthbrooding period and that some females experience gonadal recrudescence. Another possibility to be considered is that the mid-cycle decline in estradiol levels could be due to a rapid utilization of the hormone in stimulating vitellogenesis.

Rinchard *et al.* (Rinchard et al. 1993 ) Mentioned that in other teleosts such as Gudgeon (*Gobio gobio*), there was no decrease of E2 level during oocyte maturation; meanwhile this study has shown decreased E2 in some specimens of *Cyprinus carpio*.

Figure 2 shows that the highest of testosterone level was observed in summer season that this increase in testosterone in the plasma could be associated with the increase in the water temperature which occurs at the summer season. Temperature appears to be a possible cue causing testosterone to peak which leads to the gonads, and subsequently their gametes, reaching reproductive maturity.

In present results for *Cyprinus carpio*, showed that correspond with those for most teleosts fish and vertebrates, testosterone has been reported in the blood of a number of female teleosts. The slight increase of testosterone levels during oocyte development can be related to its role as precursor of 17-β estradiol synthesis, as a precursor of 17-β estradiol production, testosterone is available in the ovary for aromatization (Rinchard et al. 1993 ).

Also the highest level of progesterone hormone was observed in summer season.

During the period of summer until spring, the gonads show a relatively sharp decline in progesterone levels with some mild fluctuation corresponding with the bimodal breeding cycle. Progesterone also seems to increase in concentration as a result of the increase in water temperature that is noted during summer.

Fish are in close contact with their environment and, as a result, their physiology is influenced accordingly. However, (Sakomoto et al. 2001 ) have proposed that variations in blood parameters among fish could be affected by other variables such as the sampling technique, the capturing method, handle accuracy, the condition of captivity and the analysis techniques.
